# Factors Affecting High-Risk for Diabetes among Korean Adolescents: An Analysis Using the Eighth Korea National Health and Nutrition Examination Survey (2020)

**DOI:** 10.3390/children9081249

**Published:** 2022-08-19

**Authors:** Kyung-Sook Bang, Sang-Youn Jang, Ji-Hye Choe

**Affiliations:** Center for Human-Caring Nurse Leaders for the Future by Brain Korea 21 (BK 21) Four Project, College of Nursing, Seoul National University, Seoul 03080, Korea

**Keywords:** adolescent, diabetes mellitus, adolescent fathers, adolescent mothers, risk factors

## Abstract

The purpose of this study was to identify significant factors affecting diabetes and pre-diabetes in South Korean adolescents, including adolescents’ and parental factors. We used data on 416 Korean adolescents aged 12–18 years and their parents (302 fathers and 375 mothers) from the eighth National Health and Nutrition Examination Survey gained in 2020. The data were analyzed by descriptive statistics, *t*-test, Rao–Scott χ^2^ test, and univariate logistic regression using complex sample analysis. Among the participants, 101 adolescents (22.7%) were classified as the high-risk group for diabetes. Significant factors affecting the risk for adolescent diabetes in both sexes were higher BMI, fasting plasma glucose, hemoglobin A1c, and insulin. The father’s high degree of stress perception was only related to male adolescents, and the father’s poor subjective health status was related to females at risk for diabetes. In mothers, physician-diagnosed diabetes, fasting plasma glucose, and hemoglobin A1c were factors affecting both sexes. Results from this study can be used as preliminary data for the early detection of high-risk groups for diabetes in adolescents, and for the development of systematic health care guidelines to prevent diabetes in adolescents.

## 1. Introduction

The incidence of diabetes is steadily increasing worldwide. Korea is no exception, and the age of onset of diabetes is getting younger. According to a recent study using data from the National Health and Nutrition Examination Survey (KNHANES), the prevalence of diabetes and prediabetes in Korean children and adolescents between 2007 and 2018 was 0.298% and 7.914%, respectively [1]. Furthermore, between 2007–2009 and 2016–2018, the prevalence of diabetes and prediabetes increased approximately two-fold [1]. Diabetes leads to increased morbidity and mortality due to the development of complications affecting almost every organ in the body [2]. Although the prevalence of diabetic complications in adolescence is low, atherosclerotic vascular changes are known to occur in early adolescence [3]. In particular, adolescents with type 2 diabetes have an overall 6.15-fold increased risk of all vascular diseases compared to adolescents without diabetes [4]. Diabetes management is not limited to drug intake only; lifestyle habits are important influencing factors. The recent increase in obesity and smoking rates among adolescents, along with nutritional imbalances, are primary factors affecting the increase in diabetes among adolescents.

The International Society of Pediatric and Adolescent Diabetes (ISPAD) guidelines recommend screening using either fasting, random, or 2-h post-challenge glucose, or HbA1c [5]. According to previous studies, childhood obesity is associated with the risk of impaired fasting glucose (IFG) [6], and IFG in adolescence significantly increases later risk of type 2 diabetes and cardiovascular disease [7]. In particular, it is known that 25% of IFG develop diabetes within 3–5 years [8]. Metabolic syndrome is also a risk factor for type 2 diabetes and cardiovascular disease, and as the body mass index (BMI) of adolescents increases, the likelihood of developing metabolic syndrome also increases [9]. In addition, another study reported a positive correlation between uric acid and insulin resistance in obese children and adolescents [10]. Considering the above, it is possible to reduce the onset of diabetes in adults by the early detection and management of risk factors for diabetes such as obesity and prediabetes in adolescence.

The most common form of diabetes among adolescents is type 1 diabetes, which causes absolute insulin deficiency, but the prevalence of type 2 diabetes among adolescents is also increasing due to the recent increase in childhood obesity [11]. The etiology of type 1 diabetes is multifactorial and the specific roles for genetic susceptibility, environmental factors, the immune system, and β-cells’ pathogenic processes have not been clearly identified [5]. Adolescent type 2 diabetes has unmodifiable risk factors, such as genetic factors, being born to a mother with gestational diabetes [12], and puberty. There are also modifiable risk factors such as obesity, excessive nutritional intake, a lifestyle with reduced energy expenditure, and insulin resistance [11]. Other potentially modifiable risk factors affecting type 2 diabetes in adolescence and young adults include depressed mood [13,14,15] and sleep-related disorders [16]. The various factors involved in the onset of diabetes motivates the need for continuous research to identify risk factors for disease prevention. However, few studies have identified risk factors for diabetes based on large-scale national survey data for Korean adolescents. In addition, according to the studies previously referred to, although genetic factors play a role in the onset of diabetes, it is difficult to find a domestic study that analyzed both adolescent-related risk factors and parent-related risk factors. Therefore, it is necessary to investigate the relationship between the characteristics of the individual and the onset of diabetes, as well as conduct further research on the relationship between the characteristics of parents and the onset of diabetes in their children.

This study aims to identify significant factors for diabetes and prediabetes in adolescents aged 12 to 18 years, including risk factors related to adolescents themselves and their parents, using data from the second year of the eighth Korea National Health and Nutrition Examination Survey (KNHANES) gained in 2020.

## 2. Materials and Methods

### 2.1. Research Design

This retrospective surveillance study was designed to identify factors affecting the high-risk group for diabetes among Korean adolescents using raw data from the second year of the eighth KNHANES.

### 2.2. Data Source and Study Population

KNHANES is a nationwide survey conducted per 3-year cycle to evaluate the health and nutritional status of South Koreans aged ≥1 year. KNHANES consists of health interviews, physical examinations, and nutrition surveys [17]. In this study, the most recent health interviews and physical examinations data were used from the second year of the eighth KNHANES (2020).

The sampling design of KNHANES utilizes the rolling sampling design so that each of the three-year samples within the cycle becomes an independent probability sample representing nationwide, and the samples for each year were designed to have similar characteristics. In the second year of the eighth KNHANES, 9949 participants aged ≥1 year participated, of whom 454 were 12–18 years old. Of the 454 adolescents who participated, 416 completed the survey related to adolescent diabetes, and with their parents (302 fathers, 375 mothers) were included as final participants in this study, having completed the fasting plasma glucose (FPG) and hemoglobin A1c (HbA1c) tests. The sample of KNHANES is based on the Population and Housing Census data of South Korea, and a representative sample of the Korean population can be extracted. The sample of this study estimated the number of Korean adolescents aged 12–18 years using complex sample weights according to the guidelines of KNHANES [17]. As a result, the adolescent sample in this study represents 3.1 million Korean adolescents.

The survey was conducted with the approval of the Research Ethics Committee of the Korea Centers for Disease Control and Prevention (approval number: 2018-01-03-2C-A), and this study was approved by the institutional review committee of the researchers’ affiliated university (IRB No. E2205/004-003). This study was conducted by downloading the raw data after agreeing to the “Pledge of Implementation of Statistical Data User Compliance” and “Consent to Data User Personal Information Collection and Data Use” from the Korea Centers for Disease Control and Prevention website. The anonymity of the original data was guaranteed.

### 2.3. Study Variables

#### 2.3.1. Diabetes

The criteria for diabetes screening and diagnosis for adolescents are the same as for adults, using the recommended FPG, 2-h plasma glucose, and HbA1c tests [18]. This study used data from FPG and HbA1c tests to confirm the high-risk group for diabetes among adolescents. High-risk groups for diabetes included prediabetes (FPG 100–125 mg/dL or HbA1c 5.7–6.4%) and diabetes (FPG ≥ 126 mg/dL or HbA1c ≥ 6.5%), and the rest were classified as general groups (FPG < 100 mg/dL or HbA1c < 5.7%).

#### 2.3.2. Characteristics of Adolescents’ Variables

Adolescent-related variables were analyzed into two groups: a general group and a high-risk group for diabetes. Adolescent variables included general characteristics (sex, age, family composition, type of house, household income, smoking experience, drinking experience), BMI, psychological variables (subjective health perception, perceived stress level, depression mood experience), lifestyle variables (sleeping hours per day, sitting time per day, number of days of physical activity per week, muscle exercise days per week), and physiological and biochemical variables (systolic blood pressure (SBP), diastolic blood pressure (DBP), FPG, HbA1c, insulin, total cholesterol (TC), high-density lipoprotein-cholesterol (HDL-C), triglyceride (TG), and uric acid) were analyzed. Among the psychological variables, subjective health perception was stratified into three groups: ‘good’, ‘average’, and ‘poor’ based on the responses to questions about the usual health status [17,19]. The level of stress perception was stratified into three groups, ‘high’, ‘average’, and ‘low’, based on the responses to questions about how much stress you usually feel [17,20,21]. Among the physiological variables, blood pressure was measured by a nurse after the participant was seated in a chair and rested for 5 min. Blood pressure was measured three times in total, and in this study, the final systolic and diastolic blood pressure measurements were determined from the average values of the second and third measurements.

#### 2.3.3. Characteristics of the Parents’ Variables

The analysis of parent-related variables included BMI, medical history (physician-diagnosed diabetes, physician-diagnosed dyslipidemia), psychological variables (subjective health perception, perceived stress level), and physiological and biochemical variables (SBP, DBP, FPG, HbA1c, insulin, TC, HDL-C, TG, uric acid). The parent’s BMI (kg/m^2^) was calculated by dividing the weight by the square of the height. Diabetes and dyslipidemia were classified into two groups according to whether or not they were diagnosed by a physician. As with the adolescents, subjective health perception was stratified into ‘good’, ‘average’, and ‘poor’, and the level of stress perception was stratified into ‘high’, ‘average’, and ‘low’. Parental blood pressure variables were also measured using the average values of the second and third measurements.

### 2.4. Data Analysis

The KNHANES data were extracted with a multi-stage complex stratified cluster sample design and analyzed by the complex sample analysis method comprised of the stratification variables, clustering variables, and weights. The weights were used to increase the representation and accuracy of the estimates of the Korean population. The collected data were analyzed using SPSS version 25.0 (IBM Corp., New York, NY, USA).

The differences in general characteristics, physiological and biochemical variables of the general and high-risk groups for adolescent diabetes were analyzed by *t*-test and Rao-Scott chi-square test. To identify the adolescent and parental factors affecting the group of adolescents at high risk for diabetes, a complex sample univariate logistic regression analysis was used. Statistical significance was set at a *p*-value < 0.05.

## 3. Results

### 3.1. Comparison of General Characteristics in General and High-Risk Groups for Diabetes among Adolescents

The extracted data included 416 adolescents aged 12–18 years who completed FPG and HbA1c tests. The adolescent sample can be represented by 3.1 million Korean adolescents. Among adolescent subjects, 77.3% were in the general group, and 22.7% were in the high-risk group for diabetes. There was a significant difference in age (*p* = 0.018) between the general group and the high-risk group for diabetes (Table 1).

### 3.2. Comparison of Physiological and Biochemical Indicators in General and High-Risk Groups for Diabetes among Adolescents

The factors that showed differences in health variables in the general and the high-risk group for adolescent diabetes were SBP, FPG, HbA1c, insulin, TG, and uric acid (Table 2).

### 3.3. Factors Affecting High-Risk for Diabetes among Adolescents

#### 3.3.1. Adolescent Factors

A complex sample univariate logistic regression analysis was conducted to identify factors affecting the high risk for diabetes among adolescents (Table 3). In the total population, the prevalence of high risk for diabetes was significantly increased by BMI, subjective health perception, average weekly sleep time, SBP, FPG, HbA1c, insulin, TG, and uric acid. On the other hand, the prevalence of high risk for diabetes was lower for those aged 15–18 years, compared to those aged 12–14 years, and lower for those who exercised 1–3 days a week for about 60 min a day. In physiological and biochemical indicators, high SBP, FPG, HbA1c, insulin, TG, and uric acid were significant factors affecting high risk for adolescent diabetes.

However, there were some differences in factors related to adolescents’ high risk for diabetes in both sexes. Common factors in males and females were at the highest risk of diabetes when BMI indicated ‘obesity’ compared to ‘underweight/normal’ (Table 3). Increased FPG, HbA1c, and insulin increased the risk of diabetes among adolescents. In male adolescents, the risk of diabetes significantly decreased with increasing age and physical activity for 60 min a day. In female adolescents, average daily sleep time, SBP, TG, and uric acid were factors that significantly increased the risk of diabetes (Table 3).

#### 3.3.2. Paternal Factors

The analysis included 302 fathers of adolescents. Overall, no significant paternal factors affected the group of adolescents at high risk for diabetes. However, significant paternal factors were associated with the high-risk group for the different sexes. When the father’s degree of stress perception was ‘high’ compared to ‘low’ and ‘average’ compared to ‘low’, a high risk of diabetes in male adolescents was observed. When the father’s subjective health perception was ‘poor’ compared to ‘good’, the risk of diabetes was higher in female adolescents (Table 4).

#### 3.3.3. Maternal Factors

The analysis included 375 mothers of adolescents. In the total population, the mother’s diagnosis of diabetes, high FPG, and high HbA1c were factors affecting the high risk for diabetes among adolescents. Here too, sex differences were observed in maternal factors related to the high risk for diabetes in adolescents. Common factors in both sexes were the mother’s diagnosis of diabetes, high FPG, and high HbA1c. The factors that differed between sexes were that only in female adolescents, the mother’s subjective health perception (‘average’ compared to ‘good’ and ‘poor’ compared to ‘good’), and high TC were associated (Table 5).

## 4. Discussion

This study investigated the factors affecting the high-risk group for diabetes among adolescents using the data of the KNHANES conducted as a nationwide large-scale survey. This study confirmed that the high-risk group for diabetes was 22.7% among the estimated 3.1 million adolescents in South Korea. The prevalence of prediabetes and diabetes and the high-risk group for diabetes among Korean adolescents has been steadily increasing compared to the past [1]; the prevalence of prediabetes and diabetes among adolescents is also increasing in many developed countries [22,23,24]. Therefore, the importance of early diagnosis and prevention of adolescent diabetes is emphasized [25], and studies are being conducted to identify factors affecting adolescent diabetes [26,27].

This study analyzed the factors affecting the adolescents at high risk for diabetes and included factors of adolescents, fathers, and mothers, respectively. Among the adolescent factors, age, BMI, subjective health perception, lifestyle variables (sleeping hours per day, number of days of physical activity per week), and physiological and biochemical variables (SBP, FPG, HbA1c, insulin, TG, uric acid) were significant influencing factors.

In this study, based on the total sample of adolescents, the risk of diabetes was significantly lower as the age increased. Recently, an increase in the prevalence of type 2 diabetes among the younger generation in Korea was reported. Hong et al. [28] analyzed the prevalence of type 2 diabetes under the age of 30 years using the Korean National Health Insurance Service data over a period of 15 years (2002–2016).

It was confirmed that the prevalence of diabetes among adolescents aged 10–14 years increased by 6.39 times, and the prevalence of diabetes among adolescents aged 15–19 years increased by 5.34 times. As such, the prevalence of diabetes among adolescents in Korea is increasing at a younger age [1,28], and these results support the results of this study. Adolescents at a young age may lack perception and ability to manage their health, and self-management of diabetes risk factors may be difficult. Therefore, health guidance from parents and schoolteachers is essential.

In this study, overweight and obesity among adolescents were factors that increased the risk of diabetes in the total adolescent sample. The risk of diabetes was significantly increased in both sexes with ‘obesity’ compared to the ‘underweight/normal’ group, and the risk of diabetes was higher in females. These results can be analyzed in relation to the physical activity of adolescents. Female adolescents tend to spend more time being sedentary with less physical activity than male adolescents [29,30], and lack of physical activity increases the risk of obesity and diabetes [18]. Previous studies showed that obese children and adolescents were less physically active than the normal weight group [30], and especially during adolescence, they tended to consume fewer fruits and vegetables, while consuming more unhealthy foods such as fast food, sweet drinks, and snacks [31,32].

In addition, our research confirmed that the risk of diabetes was significantly reduced only in male adolescents when they were physically active for more than 60 min 1–3 days per week, compared to not doing any physical activity at all. This result is consistent with the finding of the American Diabetes Association (ADA) [25], which recommends that adolescents exercise at least 3 days per week for about 60 min. Physical activity maintains appropriate body weight [25] and reduces the risk of diabetes by reducing fasting glucose and HbA1c levels [33,34]. Therefore, in order to prevent diabetes among adolescents, it is necessary to educate them in the importance of a healthy diet and physical activity.

We confirmed that the risk of diabetes increases when adolescents’ subjective health perception is ‘average’ compared to ‘good’. The subjective health perception of Korean adolescents was lower in the overweight or obese group than in the normal-weight group [20], and the more stress they perceived, the less healthy they were [35], showing that adolescents know they are unhealthy when they are exposed to risk factors for diabetes. On the other hand, when adolescents felt less stressed and happier, they perceived their health levels to be high [20]. Additionally, adolescents who engage in physical activity or exercise positively accept their body image [36], and as their health perception level increases, it motivates them to practice physical activity or exercise [37]. These findings suggest that the risk of diabetes can be reduced by improving the emotional and physical health perception levels of adolescents.

Meanwhile, parent-related major factors found to have an effect on the high-risk group for diabetes were identified as the father’s degree of stress perception, parents’ subjective health perception, mother’s diabetes diagnosis, and mother’s FPG and HbA1c. In this study, it was found that the risk of diabetes increased 16.88–17.48 times only in male adolescents as the father’s stress perception increased. Previous studies showed that parental stress affects children’s stress [38,39]. However, this study found that only male adolescents were affected by the degree of the father’s stress perception. The reason can be inferred from the differences in parenting styles and communication methods between parents and children.

In the Korean society, mothers are more deeply involved in raising their children. As a result, children often interact with their mothers and spend less time with their fathers [40]. Among Korean adolescents, 47.7% answered that they spend less than 30 min with their fathers, and in particular, more male adolescents answered that they rarely talked with their fathers compared to female adolescents [40]. Previous studies have shown that when fathers communicate with their children, they strictly control their children and tend to engage in negative parenting behaviors, which increases the stress of adolescents [41]. In general, exposure to stress increases serum cortisol levels, and high cortisol levels decrease beta-cell function in the pancreas [42]. Eventually, continuous exposure to stress during adolescence leads to a risk of insulin resistance [26], which may increase the risk of diabetes. Therefore, fathers need to manage their stress and understand the importance of open and positive communication.

In addition, this study found that the parent’s poor subjective health perception was a factor that increased the risk of diabetes in female adolescents. Parents influence their children’s perception of health, eating habits, and physical activity [43]. Previous studies found that parents’ health perception was related to their children’s health intentions to control their weight and exercise and affected their healthy food intake [44,45,46]. Therefore, it is necessary for parents to understand that their health perception can affect their children’s health behavior, and to manage their own health and their children’s by identifying risk factors for diabetes in their daily eating habits and physical activity.

We also confirmed the mother’s diagnosis of diabetes and high FPG and HbA1c as factors affecting the high-risk group for diabetes among adolescents. A family history of diabetes is one of the primary risk factors for diabetes [18]. Previous studies have shown that mothers with a family history of diabetes pose a higher risk of diabetes development in their children than fathers [47], and children of mothers with a family history of diabetes have a higher risk of poor glycemic control [48]. These findings support the results of this study. That is, the mother’s diagnosis of diabetes, high FPG, and high HbA1c were factors that had a significant effect on the children’s high-risk group for diabetes. Therefore, our findings suggest that knowing the mother’s health status and family history can be used as a basis for screening high-risk groups for diabetes in adolescents.

This study has the strength to generalize the research results by analyzing representative subjects using large-scale national survey data. Moreover, the study is meaningful in that we analyzed and identified high-risk factors for diabetes in adolescents considering both the characteristics of adolescents and their parents, and prepared basic data for early detection and prevention of adolescent diabetes.

As for the limitations, first, since the results of this study only used data from the second year of the eighth KNHANES, there was a limitation in the number of participants to be analyzed. Therefore, we suggest expanding to research that integrates and analyzes the first and second year data, including the third year data to be released in the future. Second, since the KNHANES questionnaire did not differentiate between type 1 and type 2 diabetes, we could not distinguish the factors influencing type 1 or type 2 diabetes. Therefore, KNHANES needs to consider additional data collection methods that can diagnose type 1 and type 2 diabetes differently. Then, further studies are needed to identify what influencing factors are involved in each type 1 and type 2 diabetes. Nevertheless, this study is meaningful in that it identified factors affecting the high-risk group for diabetes among adolescents.

## 5. Conclusions

This study was conducted to identify factors affecting the high-risk groups for diabetes among Korean adolescents using data from the second year of the eighth KNHANES, and to contribute to establishing an intervention strategy for the early detection and prevention of adolescent diabetes in the high-risk groups.

In this study, the high-risk group for adolescent diabetes was 22.7% among South Korean adolescents, and the high-risk groups for diabetes showed increased physiological and biochemical indicators (SBP, FPG, HbA1c, insulin, TG, uric acid). Among the factors significantly predicting the high-risk groups of adolescent diabetes were higher BMI, FPG, HbA1c, and insulin in both sexes. A difference in the father’s high level of stress perception was associated with male adolescents, and the father’s poor subjective health perception was associated with females at risk for diabetes. The mother’s diagnosis of diabetes, high FPG, and HbA1c were significant factors that increased the risk of diabetes in both sexes of the adolescents.

The results of this study confirmed the importance of health management strategies of adolescents and their parents. Moreover, this study provided basic data on the characteristics of high-risk groups for adolescent diabetes and suggested the necessity to develop an intervention program for the early prevention of diabetes centered on adolescent diabetes high-risk groups.

## Figures and Tables

**Table 1:** Comparison of General Characteristics in General and High-risk Groups for Diabetes among Adolescents (*N* = 416).

Variables	Categories	General Group (n = 315, 2.4 m *)	High-Risk Group (n = 101, 0.7 m *)	Total (n = 416, 3.1 m *)	Rao–Scott χ^2^ (*p*)
		n (%)	n (%)	n (%)	
Sex	Male	173 (75.5)	63 (24.5)	236 (100.0)	0.73 (0.394)
	Female	142 (79.4)	38 (20.6)	180 (100.0)	
Age (years)	12–14	143 (71.2)	62 (28.8)	205 (100.0)	5.78 (0.018)
	15–18	172 (81.7)	39 (18.3)	211 (100.0)	
Family composition ^†^	2 generations	272 (78.5)	82 (21.5)	354 (100.0)	0.26 (0.612)
	3 generations	31 (75.2)	12 (24.8)	43 (100.0)	
Type of house ^†^	Detached house	38 (69.1)	24 (30.9)	62 (100.0)	1.42 (0.243)
	Apartment	239 (79.3)	64 (20.7)	303 (100.0)	
	Other	37 (73.3)	13 (26.7)	50 (100.0)	
Household income ^†^	Low	19 (75.7)	8 (24.3)	27 (100.0)	0.40 (0.666)
	Middle	203 (76.0)	70 (24.0)	273 (100.0)	
	High	92 (80.4)	23 (19.6)	115 (100.0)	
Smoking experience	Yes	29 (84.4)	8 (15.6)	37 (100.0)	1.08 (0.300)
	No	286 (76.6)	93 (23.4)	379 (100.0)	
Drinking experience	Yes	68 (77.8)	22 (22.2)	90 (100.0)	0.02 (0.882)
	No	247 (77.1)	79 (22.9)	326 (100.0)	

* Weighed sample size was estimated population represented in millions. ^†^ Excluding non-response.

**Table 2:** Comparison of Physiological and Biochemical Indicators in General and High-Risk Groups for Diabetes among Adolescents (*N* = 416).

Variables	Categories	General Group (n = 315, 2.4 m *)	High-Risk Group (n = 101, 0.7 m *)	Total (n = 416, 3.1 m *)	Rao–Scott χ^2^ or t (*p*)
		n (%) or M ± SD	n (%) or M ± SD	n (%) or M ± SD	
BMI ^†^	Underweight/Normal	242 (83.2)	55 (16.8)	297 (100.0)	12.84 (<0.001)
	Overweight	30 (65.5)	15 (34.5)	45 (100.0)	
	Obesity	41 (57.2)	30 (42.8)	71 (100.0)	
Subjective health perception	Good	201 (83.2)	48 (16.8)	249 (100.0)	4.85 (0.009)
	Average	102 (68.6)	46 (31.4)	148 (100.0)	
	Poor	12 (66.9)	7 (33.1)	19 (100.0)	
Perceived stress level	High	80 (82.1)	21 (17.9)	101 (100.0)	1.06 (0.349)
	Average	172 (74.6)	62 (25.4)	234 (100.0)	
	Low	63 (79.0)	18 (21.0)	81 (100.0)	
Depressive mood experience	Yes	18 (75.9)	7 (24.1)	25 (100.0)	0.02 (0.882)
	No	297 (77.4)	94 (22.6)	391 (100.0)	
Sleeping hours/day		6.72 ± 0.09	7.10 ± 0.13	6.81 ± 0.07	−2.09 (0.038)
Sitting time/day		11.40 ± 0.28	10.97 ± 0.34	11.30 ± 0.23	1.01 (0.316)
Physical activity days/week	Not at all	198 (74.5)	73 (25.5)	271 (100.0)	2.93 (0.065)
	1–3/week	81 (86.6)	15 (13.4)	96 (100.0)	
	4–7/week	36 (72.6)	13 (27.4)	49 (100.0)	
Muscle exercise days/week	Not at all	183 (77.6)	57 (22.4)	240 (100.0)	0.12 (0.887)
	1–3/week	87 (77.9)	29 (22.1)	116 (100.0)	
	4–7/week	45 (74.4)	15 (25.6)	60 (100.0)	
SBP (mmHg)		109.46 ± 0.84	112.73 ± 1.07	110.22 ± 0.68	−2.39 (0.018)
DBP (mmHg)		69.44 ± 0.57	70.40 ± 0.96	69.66 ± 0.51	−0.90 (0.368)
FPG (mg/dL)		89.37 ± 0.30	101.14 ± 2.25	92.04 ± 0.65	−5.28 (<0.001)
HbA1c (%)		5.30 ± 0.02	5.68 ± 0.07	5.39 ± 0.02	−5.83 (<0.001)
Insulin (uIU/mL)		14.24 ± 0.70	28.83 ± 3.33	17.56 ± 0.94	−4.13 (<0.001)
TC (mg/dL)		161.47 ± 1.52	167.09 ± 3.52	162.75 ± 1.53	−1.55 (0.123)
HDL-C (mg/dL)		51.39 ± 0.52	49.33 ± 1.02	50.92 ± 0.48	1.89 (0.059)
TG (mg/dL)		90.62 ± 3.56	112.54 ± 7.93	95.60 ± 3.39	−2.56 (0.011)
Uric acid (mg/dL)		5.46 ± 0.08	5.92 ± 0.17	5.57 ± 0.08	−2.47 (0.014)

* Weighed sample size was estimated in millions, ^†^ Excluding non-response; BMI, body mass index; SBP, systolic blood pressure; DBP, diastolic blood pressure; FPG, fasting plasma glucose; HbA1c, hemoglobin A1c; TC, total cholesterol; HDL-C, high-density lipoprotein-cholesterol; TG, triglyceride.

**Table 3:** Adolescent Factors Affecting the High-risk Group for Adolescent Diabetes.

Variables (Reference)	Categories	Total (n = 416, 3.1 m *)		Males (n = 236, 1.6 m *)		Females (n = 180, 1.4 m *)	
		OR (95% CI)	*p*	OR (95% CI)	*p*	OR (95% CI)	*p*
Age (12–14 years)	15–18 years	0.55 (0.34, 0.90)	0.019	0.44 (0.24, 0.83)	0.012	1.39 (0.64, 3.04)	0.406
Household income (High)	Middle	1.29 (0.71, 2.34)	0.398	1.38 (0.57, 3.35)	0.479	1.30 (0.49, 3.46)	0.596
	Low	1.32 (0.47, 3.67)	0.596	1.58 (0.38, 6.65)	0.531	1.28 (0.28, 5.83)	0.751
Smoking experience (No)	Yes	0.60 (0.23, 1.59)	0.305	0.80 (0.29, 2.22)	0.662	0.29 (0.03, 2.45)	0.254
Drinking experience (No)	Yes	0.96 (0.55, 1.67)	0.882	0.93 (0.46, 1.90)	0.847	1.01 (0.39, 2.58)	0.991
BMI (Underweight/Normal)	Overweight	2.62 (1.30, 5.26)	0.007	2.34 (1.01, 5.43)	0.049	2.76 (0.69, 10.98)	0.149
	Obesity	3.71 (2.08, 6.61)	<0.001	2.44 (1.11, 5.36)	0.027	6.98 (3.20, 15.22)	<0.001
Subjective health perception (Good)	Average	2.26 (1.37, 3.75)	0.002	2.20 (1.21, 4.01)	0.010	2.44 (0.99, 6.03)	0.054
	Poor	2.45 (0.80, 7.53)	0.117	2.59 (0.68, 9.85)	0.160	2.27 (0.32, 16.17)	0.411
Perceived stress level (Low)	High	0.82 (0.35, 1.92)	0.648	1.34 (0.46, 3.93)	0.595	0.39 (0.09, 1.80)	0.226
	Average	1.28 (0.64, 2.58)	0.484	1.08 (0.49, 2.41)	0.847	1.59 (0.53, 4.81)	0.407
Depressive mood experience (No)	Yes	1.09 (0.36, 3.32)	0.882	1.68 (0.41, 6.89)	0.472	0.87 (0.15, 4.91)	0.870
Sleeping hours/day		1.19 (1.00, 1.40)	0.044	1.13 (0.92, 1.38)	0.253	1.25 (1.01, 1.54)	0.038
Sitting time/day		0.97 (0.88, 1.08)	0.576	1.00 (0.87, 1.14)	0.953	0.94 (0.80, 1.10)	0.400
Physical activity days/week (Not at all)	1–3/week	0.45 (0.24, 0.85)	0.014	0.37 (0.17, 0.81)	0.013	0.48 (0.15, 1.54)	0.213
	4–7/week	1.11 (0.49, 2.48)	0.807	0.82 (0.29, 2.30)	0.701	1.61 (0.33, 7.76)	0.551
Muscle exercise days/week (Not at all)	1–3/week	0.98 (0.54, 1.79)	0.957	1.39 (0.68, 2.85)	0.360	0.42 (0.15, 1.19)	0.101
	4–7/week	1.19 (0.54, 2.62)	0.658	1.14 (0.48, 2.73)	0.762	1.92 (0.32, 11.34)	0.470
SBP (mmHg)		1.03 (1.00, 1.05)	0.021	1.00 (0.98, 1.03)	0.829	1.08 (1.03, 1.12)	0.002
DBP (mmHg)		1.01 (0.99, 1.04)	0.367	1.00 (0.97, 1.03)	0.867	1.04 (0.99, 1.09)	0.166
FPG (mg/dL)		1.25 (1.16, 1.35)	<0.001	1.36 (1.23, 1.50)	<0.001	1.18 (1.06, 1.30)	0.002
HbA1c (%)		1.94 (1.61, 2.34)	<0.001	1.75 (1.41, 2.18)	<0.001	2.37 (1.61, 3.51)	<0.001
Insulin (uIU/mL)		1.05 (1.03, 1.08)	<0.001	1.04 (1.02, 1.07)	0.002	1.07 (1.03, 1.10)	<0.001
TC (mg/dL)		1.01 (1.00, 1.02)	0.108	1.01 (1.00, 1.02)	0.159	1.01 (0.99, 1.02)	0.321
HDL-C (mg/dL)		0.98 (0.95, 1.00)	0.073	0.98 (0.95, 1.01)	0.174	0.97 (0.93, 1.02)	0.274
TG (mg/dL)		1.01 (1.00, 1.01)	0.007	1.01 (1.00, 1.01)	0.057	1.01 (1.00, 1.01)	0.037
Uric acid (mg/dL)		1.02 (1.01, 1.04)	0.013	1.02 (0.98, 1.05)	0.375	1.06 (1.01, 1.12)	0.020

* Weighed sample size was estimated in millions; OR, odds ratio; CI, confidence interval; BMI, body mass index; SBP, systolic blood pressure; DBP, diastolic blood pressure; FPG, fasting plasma glucose; HbA1c, hemoglobin A1c; TC, total cholesterol; HDL-C, high-density lipoprotein-cholesterol; TG, triglyceride.

**Table 4:** Paternal Factors Affecting the High-risk Group for Adolescent Diabetes.

Variables (Reference)		Categories	Total (n = 416, 3.1 m *)		Males (n = 236, 1.6 m *)		Females (n = 180, 1.4 m *)	
			OR (95% CI)	*p*	OR (95% CI)	*p*	OR (95% CI)	*p*
Paternal factor (n = 302)	BMI (kg/m^2^)		1.06 (0.97, 1.15)	0.189	1.11 (0.99, 1.23)	0.066	0.97 (0.86, 1.10)	0.635
	Physician-diagnosed diabetes (No)	Yes	2.68 (0.98, 7.34)	0.054	2.95 (0.58, 15.08)	0.191	2.63 (0.74, 9.34)	0.134
	Physician-diagnosed dyslipidemia (No)	Yes	1.55 (0.75, 3.21)	0.236	1.57 (0.61, 4.06)	0.350	1.54 (0.48, 4.91)	0.463
	Subjective health perception (Good)	Average	1.49 (0.71, 3.15)	0.289	1.09 (0.43, 2.74)	0.857	2.82 (0.79, 10.08)	0.110
		Poor	2.27 (0.76, 6.72)	0.139	1.02 (0.19, 5.46)	0.985	7.32 (1.95, 27.51)	0.004
	Perceived stress level (Low)	High	1.34 (0.30, 6.01)	0.703	16.88 (2.15, 132.66)	0.008	0.24 (0.03, 2.04)	0.187
		Average	1.74 (0.43, 6.93)	0.432	17.48 (2.23, 136.80)	0.007	0.59 (0.12, 2.93)	0.515
	SBP (mmHg)		1.00 (0.98, 1.02)	0.815	1.01 (0.98, 1.03)	0.630	0.98 (0.94, 1.02)	0.247
	DBP (mmHg)		1.01 (0.97, 1.05)	0.696	1.04 (1.00, 1.09)	0.077	0.95 (0.89, 1.02)	0.130
	FPG (mg/dL)		1.01 (1.00, 1.03)	0.149	1.01 (0.99, 1.03)	0.517	1.02 (1.00, 1.04)	0.145
	HbA1c (%)		1.03 (0.99, 1.08)	0.182	1.03 (0.97, 1.09)	0.344	1.03 (0.97, 1.10)	0.312
	Insulin (uIU/mL)		0.98 (0.93, 1.03)	0.425	0.99 (0.92, 1.06)	0.770	0.96 (0.89, 1.03)	0.238
	TC (mg/dL)		1.00 (0.99, 1.01)	0.647	1.00 (0.99, 1.01)	0.884	1.01 (0.99, 1.02)	0.421
	HDL-C (mg/dL)		0.99 (0.97, 1.01)	0.373	0.97 (0.94, 1.00)	0.076	1.01 (0.98, 1.05)	0.389
	TG (mg/dL)		1.00 (1.00, 1.00)	0.201	1.00 (1.00, 1.01)	0.116	1.00 (1.00, 1.00)	0.958
	Uric acid (mg/dL)		1.01 (0.99, 1.04)	0.324	0.99 (0.95, 1.02)	0.465	1.07 (1.03, 1.12)	0.001

* Weighed sample size was estimated in millions; OR, odds ratio; CI, confidence interval; BMI, body mass index; SBP, systolic blood pressure; DBP, diastolic blood pressure; FPG, fasting plasma glucose; HbA1c, hemoglobin A1c; TC, total cholesterol; HDL-C, high-density lipoprotein-cholesterol; TG, triglyceride.

**Table 5:** Maternal Factors Affecting the High-Risk Group for Adolescent Diabetes.

Variables (Reference)		Categories	Total (n = 416, 3.1 m *)		Males (n = 236, 1.6 m *)		Females (n = 180, 1.4 m *)	
			OR (95% CI)	*p*	OR (95% CI)	*p*	OR (95% CI)	*p*
Maternal factor (n = 375)	BMI (kg/m^2^)		1.05 (0.99, 1.12)	0.136	1.06 (0.96, 1.17)	0.251	1.06 (0.98, 1.13)	0.139
	Physician-diagnosed diabetes (No)	Yes	10.02 (2.83, 35.51)	<0.001	14.44 (1.23, 170.16)	0.034	9.07 (1.44, 57.28)	0.019
	Physician-diagnosed dyslipidemia (No)	Yes	1.51 (0.61, 3.77)	0.373	1.22 (0.33, 4.49)	0.765	1.99 (0.58, 6.88)	0.275
	Subjective health perception (Good)	Average	1.01 (0.53, 1.94)	0.980	0.52 (0.22, 1.25)	0.144	3.87 (1.35, 11.10)	0.012
		Poor	1.52 (0.58, 4.00)	0.399	0.95 (0.29, 3.17)	0.932	4.86 (1.13, 21.01)	0.034
	Perceived stress level (Low)	High	0.80 (0.28, 2.24)	0.664	0.93 (0.22, 3.84)	0.915	0.59 (0.13, 2.83)	0.511
		Average	0.93 (0.36, 2.41)	0.877	0.80 (0.22, 2.98)	0.739	1.11 (0.26, 4.69)	0.891
	SBP (mmHg)		1.00 (0.98, 1.02)	0.954	0.99 (0.96, 1.02)	0.617	1.01 (0.98, 1.04)	0.407
	DBP (mmHg)		0.99 (0.96, 1.02)	0.626	1.00 (0.95, 1.04)	0.924	0.99 (0.95, 1.04)	0.688
	FPG (mg/dL)		1.03 (1.01, 1.04)	0.001	1.03 (1.00, 1.07)	0.030	1.02 (1.00, 1.04)	0.019
	HbA1c (%)		1.11 (1.05, 1.17)	<0.001	1.12 (1.02, 1.24)	0.024	1.11 (1.04, 1.18)	0.003
	Insulin (uIU/mL)		1.00 (0.95, 1.05)	0.992	0.99 (0.92, 1.07)	0.861	1.01 (0.95, 1.08)	0.690
	TC (mg/dL)		1.00 (0.99, 1.01)	0.603	1.01 (1.00, 1.02)	0.321	0.98 (0.97, 1.00)	0.028
	HDL-C (mg/dL)		1.00 (0.97, 1.02)	0.747	1.01 (0.98, 1.05)	0.438	0.97 (0.94, 1.00)	0.056
	TG (mg/dL)		1.00 (0.99, 1.00)	0.357	1.00 (0.99, 1.00)	0.485	1.00 (0.99, 1.01)	0.659
	Uric acid (mg/dL)		0.98 (0.94, 1.01)	0.229	0.98 (0.95, 1.02)	0.412	0.96 (0.90, 1.04)	0.311

* Weighed sample size was estimated in millions; OR, odds ratio; CI, confidence interval; BMI, body mass index; SBP, systolic blood pressure; DBP, diastolic blood pressure; FPG, fasting plasma glucose; HbA1c, hemoglobin A1c; TC, total cholesterol; HDL-C, high-density lipoprotein-cholesterol; TG, triglyceride.

## Data Availability

Not applicable.
